# Coinfections in wildlife: Focus on a neglected aspect of infectious disease epidemiology

**DOI:** 10.1371/journal.ppat.1008790

**Published:** 2020-09-03

**Authors:** Axel O. G. Hoarau, Patrick Mavingui, Camille Lebarbenchon

**Affiliations:** Université de La Réunion, Processus Infectieux en Milieu Insulaire Tropical, INSERM 1187, CNRS 9192, IRD 249, Saint Denis, Réunion Island, France; University of Wisconsin Medical School, UNITED STATES

Emerging infectious diseases represent a global and major health problem. The understanding of biological processes involved in the transmission and evolution of infectious agents in host reservoirs is critical [1]. Disease ecology has, therefore, become an important area of research, aiming at investigating the interactions between infectious agents, their hosts, and environments. Interactions between infectious agents exploiting the same vertebrate host at the same time (coinfections) can affect disease outcomes and transmissibility. In this paper, we review this point, as this could have significant consequences on zoonoses emergence.

## Are coinfections common?

**Fig 1:**
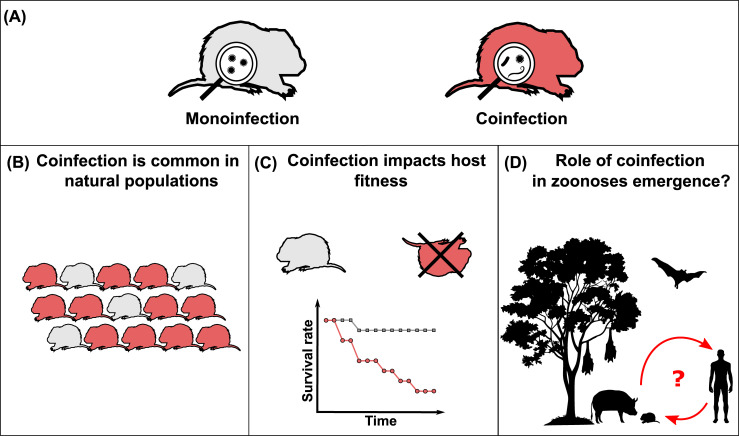
The importance of coinfection in wild hosts. (A) Coinfections are defined by the presence of at least two genetically different infectious agents in the same host: infectious agents of different taxonomic levels (e.g., bacterium and virus) but also genetic variants of the same infectious agent (e.g., virus genotypes). (B) Coinfection is the rule in all living communities and prevalence can be high in vertebrate animals. (C) Coinfection can impact host fitness. (D) The importance of coinfection in zoonoses emergence processes remains to be fully assessed.

Coinfection (or co-infection), which can refer to simultaneous infection, mixed infection, multiple infections, concomitant infection, concurrent infection, polyinfection, polyparasitism, and multiple parasitisms [2], defines the occurrence of at least two genetically different infectious agents in the same host (Fig 1A) [3]. This definition, therefore, includes infectious agents of different taxonomic levels (e.g., bacterium and virus) and also genetic variants of the same infectious agent (e.g., virus genotypes) [3]. Coinfections have been mainly studied in humans, with a particular emphasis on macroparasite helminths [3,4]. The presence of helminth eggs from multiple species has even been reported in human remains and coprolites recovered from many prehistoric sites and analyzed by microscopy [5]. About 30% of human infections may actually be coinfections, and this rate could reach up to 80% in some human communities [4,6]. Recent studies have focused on other living organisms such as plants, vertebrate, and invertebrate animals and have demonstrated that coinfection is indeed the rule rather than the exception (Fig 1B) [2–4,7]. For example, coinfection by protozoa (*Eimeria* sp., *Entamoeba* sp., *Giardia* sp. and *Cryptosporidium* sp.) and by protozoa and helminths (e.g., Ancylostomatidae, *Vampirolepsis nana*) in Brazilian bats can reach 22% in *Molossus molossus*, 25% in *Myotis lavali*, and 36% in *Noctilio albiventris* [8]. In field voles (*Microtus agrestis*), the prevalence of coinfected individuals with *Babesia microti*, Cowpox virus, *Anaplasma phagocytophilum*, and *Bartonella spp*., can reach up to 79% of tested animals [9].

## How do infectious agents interact?

Coinfection not only reflects the simultaneous presence of multiple infectious agents in a given host but also involves complex interactions between them. The type of interactions within a community of infectious agents exploiting the same host can be direct, for example, via physical interference or competition for resources, or indirect, such as through immunological pathways or the production of chemical compounds [10–17]. The outcome of these interactions can be positive (synergistic), in which the presence of one infectious agent may facilitate infection by other infectious agents; negative (antagonistic), when the presence of one infectious agent inhibits infection or replication; or neutral when the presence of one infectious agent does not affect the infection by other infectious agents [6,14,16]. In field voles, infection patterns have been shown to be highly conditioned by a complex web of interactions, depending on the presence or absence of the other infectious agents [9]. Both positive interactions (e.g., Cowpox virus and *Bartonella* bacteria) and negative interactions (e.g., *Anaplasma phagocytophilum* and *Babesia microti*) contribute to the global web of relationships. Some infectious agents seem to directly compete for blood as a resource (*Babesia microti* and *Bartonella* bacteria) and others indirectly through immunomodulatory effects (e.g., *Anaplasma phagocytophilum* and Cowpox virus) [9]. The cocirculation of paramyxovirus species in Australian flying foxes (Genus *Pteropus*) also seems to be driven by conditional associations. Indeed, at the sample level, several commonly detected species in natural populations seem to positively interact (e.g., Teviot virus and Hendra virus or Yeppoon virus) and may suggest immunomodulatory effects, such as activation of latent infections; whereas other species rarely detected together could interact negatively (e.g., Hendra virus and Yeppoon virus) and may suggest competition [18].

## What are the effects of coinfections on host fitness?

Coinfections have a large range of effects on host fitness, and this emanates from the interactions taking place among the infectious agents (Fig 1C) [6,14,16,17]. The consequences are not simply the sum of the effects caused by each infectious agent, but the result of a complex combination of known and novel effects affecting key epidemiological parameters, often resulting in more pronounced effects than the individual infections alone [6,11,19]. Coinfections can have negative consequences, from abnormal symptoms for a given disease to accelerated death [6]. For example, mice (*Mus musculus*) coinfected by gastrointestinal helminths and respiratory bacteria are able to chronically shed a larger number of helminth eggs than monoinfected individuals [20]. African buffaloes (*Syncerus caffer*) coinfected by gastrointestinal nematodes and *Mycobacterium bovis* face an accelerated mortality [21]. Coinfections can also have beneficial effects for the host, for instance, by taking advantage of antagonistic interactions occurring between infectious agents. For example, gray treefrogs (*Hyla versicolor*), Northern leopard frogs (*Lithobates pipiens*), and spring peepers (*Pseudacris crucifer*) coinfected with helminth *Echinoparyphium* and Ranavirus, have lower viral load than individuals only infected by the virus, suggesting that macroparasite infection can reduce microparasite infection, possibly through crossreactive immunity [22]. Pekin ducks (*Anas platyrhynchos var*. *domestica*) coinfected with Newcastle disease virus and low-pathogenic avian influenza virus, face a decrease of influenza virus shedding and transmission, suggesting viral interference between the two infectious agents [23]. “Forced” coinfection can also be beneficial for the host, for example, phage therapy, defined as the use of bacteriophage as treatment against targeted bacteria, is based on antagonistic interactions [6,24]. More broadly, beyond the direct consequences on host fitness, simultaneous infections can strongly affect the dynamics of infectious agents by modifying host susceptibility, infection probabilities, or transmission rates [6].

## Why should we investigate coinfection in wild hosts?

Disease ecology studies have highlighted the key role of environmental and biological factors in spatial and temporal infection dynamics. For example, seasonal changes in social behavior (e.g., grouping of animals at water sources during dry season) or changes in population structure (e.g., increase of the amount of immunologically naïve juveniles during breeding season) affect transmission opportunities and, therefore, infection dynamics in many species [25]. However, although coinfections are widespread in living communities, and demonstrate epidemiological consequences, most studies have been limited to the investigation of a one host and one infectious agent system, largely because of the complexity of natural systems [13]. Although challenging, future studies need to integrate the natural diversity of infectious agents among living communities and fully investigate interaction networks, by considering infectious agents from different taxonomic levels (e.g., virus, bacteria, protozoa, and helminths) and by investigating a broad variety of samples for each host (e.g., feces, blood, and urine) through longitudinal studies. It has been illustrated that considering only a part of the infectious agents community when assessing the effects on host fitness is biased [26]. These investigations can be performed at individual, population, or community levels in order to identify the outcomes on infectious agent dynamics [13]. Given that two-thirds of emerging infectious diseases are zoonoses, with nearly 70% originating from wildlife, better knowledge of the interactions of infectious agents in wild reservoirs will provide key insight for the understanding and management of spillover processes [27,28]. In particular, the role of coinfections in wild and also domestic hosts (e.g., livestock), in the emergence of zoonoses, remains to be fully assessed (Fig 1D).

## How coinfections in wild animals can be studied?

Coinfections in wild animals can be investigated using classical approaches based on sample collection, infectious agent detection, and analysis of the results. Sample collection can be achieved through cross-sectional or longitudinal studies [6]. Cross-sectional studies provide information on the co-occurrence of infectious agents at the time of sampling, whereas longitudinal studies provide a more detailed information in the infection dynamics in individual hosts and communities over time. However, most studies designed to investigate coinfection in wildlife are restricted to limited “niches” (e.g., blood, saliva, urine, and feces), and collected samples are analyzed with targeted assays (e.g. PCR and serology), therefore, offering few opportunities to investigate generic coinfection [4,11]. The development and improvement of new approaches such as metagenomics, next generation sequencing, and bioinformatics, provides a method to simultaneously describe a large number of pathogens without previous knowledge and a priori [13,29,30] and allow to share an increasing amount of data with the scientific community, therefore, offering new insights compared to traditional methodologies. Regarding the analytical approach, statistical tests such as chi-squared test can be used to quickly examine co-occurrence but often with limited assumptions concerning interactions and their consequences [6]. Many other statistical tests, mathematical models, and ecological theories have been developed to better infer interactions, although approaches vary depending on study designs and infectious agents [10,14–16]. Field studies can also be associated with experimental approaches such as captive studies (with wild animals) or mesocosms (artificial ecosystems) [14,22].

## Conclusion

Coinfections are recognized to be the rule in all living communities and have consequences on both host fitness and infectious agent epidemiology. The emergence of infectious diseases associated with wild hosts highlights the need for a better knowledge on the factors and mechanisms involved in the epidemiology of infectious diseases in natural populations. Although challenging, both theoretical and experimental tools are available. This provides a unique opportunity to gain better fundamental knowledge on infectious agent interactions and also to investigate the role and consequences of coinfections in the emergence of zoonoses.
